# Trap Array Configuration Influences Estimates and Precision of Black Bear Density and Abundance

**DOI:** 10.1371/journal.pone.0111257

**Published:** 2014-10-28

**Authors:** Clay M. Wilton, Emily E. Puckett, Jeff Beringer, Beth Gardner, Lori S. Eggert, Jerrold L. Belant

**Affiliations:** 1 Carnivore Ecology Laboratory, Forest and Wildlife Research Center, Mississippi State University, Mississippi State, MS, United States of America; 2 Division of Biological Sciences, University of Missouri, Columbia, MO, United States of America; 3 Missouri Department of Conservation, Columbia, MO, United States of America; 4 North Carolina State University, Department of Forestry and Environmental Resources, Raleigh, NC, United States of America; University of Zurich, Switzerland

## Abstract

Spatial capture-recapture (SCR) models have advanced our ability to estimate population density for wide ranging animals by explicitly incorporating individual movement. Though these models are more robust to various spatial sampling designs, few studies have empirically tested different large-scale trap configurations using SCR models. We investigated how extent of trap coverage and trap spacing affects precision and accuracy of SCR parameters, implementing models using the R package *secr*. We tested two trapping scenarios, one spatially extensive and one intensive, using black bear (*Ursus americanus*) DNA data from hair snare arrays in south-central Missouri, USA. We also examined the influence that adding a second, lower barbed-wire strand to snares had on quantity and spatial distribution of detections. We simulated trapping data to test bias in density estimates of each configuration under a range of density and detection parameter values. Field data showed that using multiple arrays with intensive snare coverage produced more detections of more individuals than extensive coverage. Consequently, density and detection parameters were more precise for the intensive design. Density was estimated as 1.7 bears per 100 km^2^ and was 5.5 times greater than that under extensive sampling. Abundance was 279 (95% CI = 193–406) bears in the 16,812 km^2^ study area. Excluding detections from the lower strand resulted in the loss of 35 detections, 14 unique bears, and the largest recorded movement between snares. All simulations showed low bias for density under both configurations. Results demonstrated that in low density populations with non-uniform distribution of population density, optimizing the tradeoff among snare spacing, coverage, and sample size is of critical importance to estimating parameters with high precision and accuracy. With limited resources, allocating available traps to multiple arrays with intensive trap spacing increased the amount of information needed to inform parameters with high precision.

## Introduction

Knowledge of population size and spatial distribution is important for protection of threatened or endangered species [1]–[3], and management of harvested animal populations [4], [5]. Estimates of species’ abundance or density are useful as a baseline for developing protected areas [6], [7], prioritizing conservation actions [8], [9], and allocating harvest quotas [10]. However, large mammals often persist at low densities over large areas, are not uniformly distributed, and have large home ranges [11]–[13]. These characteristics may undermine abundance estimation and hinder subsequent conservation efforts [14]–[15].

Capture-recapture methods are often used to estimate density and abundance of rare or elusive carnivores [2], [15], [16]. Remote collection of DNA samples (e.g., hair, feces) enables researchers to sample wide geographic areas [17], [18], and has become almost universal for bear (*Ursus* spp.) capture-recapture studies [19], [20]. Nonetheless, trap configurations that do not adequately reflect population distributions and individual variation in space use may limit precise and accurate estimates of density and abundance [15], [21].

The spatial nature of sampling designs (e.g., trap distribution) and wildlife populations (e.g., home range distribution) are important components of estimating animal abundance [22]–[24]. Non-spatial capture-recapture models often require study designs to cover several times the area of an individual home range [25], while maintaining trap spacing narrow enough to ensure individuals have nonzero and homogenous capture probabilities [26], [27]. However, for species with large home ranges and individual movements, logistical constraints may require a tradeoff between extensive coverage of a study area with wide trap spacing or intensive coverage of a portion of the study area with close spacing [28], [29].

Spatial capture-recapture (SCR) models explicitly include animal movement and trap distribution, and therefore reduces constraints placed on sampling wide ranging species over large areas [30], [31]. Moreover, SCR defines a spatial point process model to estimate the home range (i.e., activity) centers of individuals detected, eliminating the need for ad hoc estimates of the effective sampling area [32]. Therefore, SCR models address a primary source of heterogeneity inherent in most carnivore populations by addressing unequal exposure to traps and edge effects [33], [34]. Simulations of SCR parameter estimates from black bear trapping configurations were unbiased when movement was at least half the distance between traps and when trap coverage was similar to the extent of movement [29], [35]. Although SCR models are robust to unequal trap exposure and appear flexible to various spatial trapping designs [34], few studies have empirically tested the efficacy of SCR models using different large-scale trap array configurations.

The large home ranges of bears and constraints to large-scale sampling often preclude adequate coverage of individual space use [36]. We tested a spatially extensive and intensive trapping scenario to compare how trap coverage and spacing affects precision of SCR parameter estimates using black bear (*Ursus americanus*) DNA encounter history data from hair snare arrays. To generalize the findings of our spatial sampling configurations and evaluate their accuracy, we also conducted simulations to measure bias under realistic densities and detection probabilities for large carnivores. We also consider effects of snare design on detections and provide insights towards implementing large scale capture-recapture sampling designs for SCR models for low density, wide ranging species.

## Methods

### Ethics Statement

All sampling methods complied with guidelines established by the American Society of Mammalogists and this study was approved by the Institutional Animal Care and Use Committee protocol (approval 10-037) at Mississippi State University. Sampling locations and procedures were approved by the Missouri Department of Conservation and sampling procedures did not involve endangered species.

### Study area

**Figure 1:**
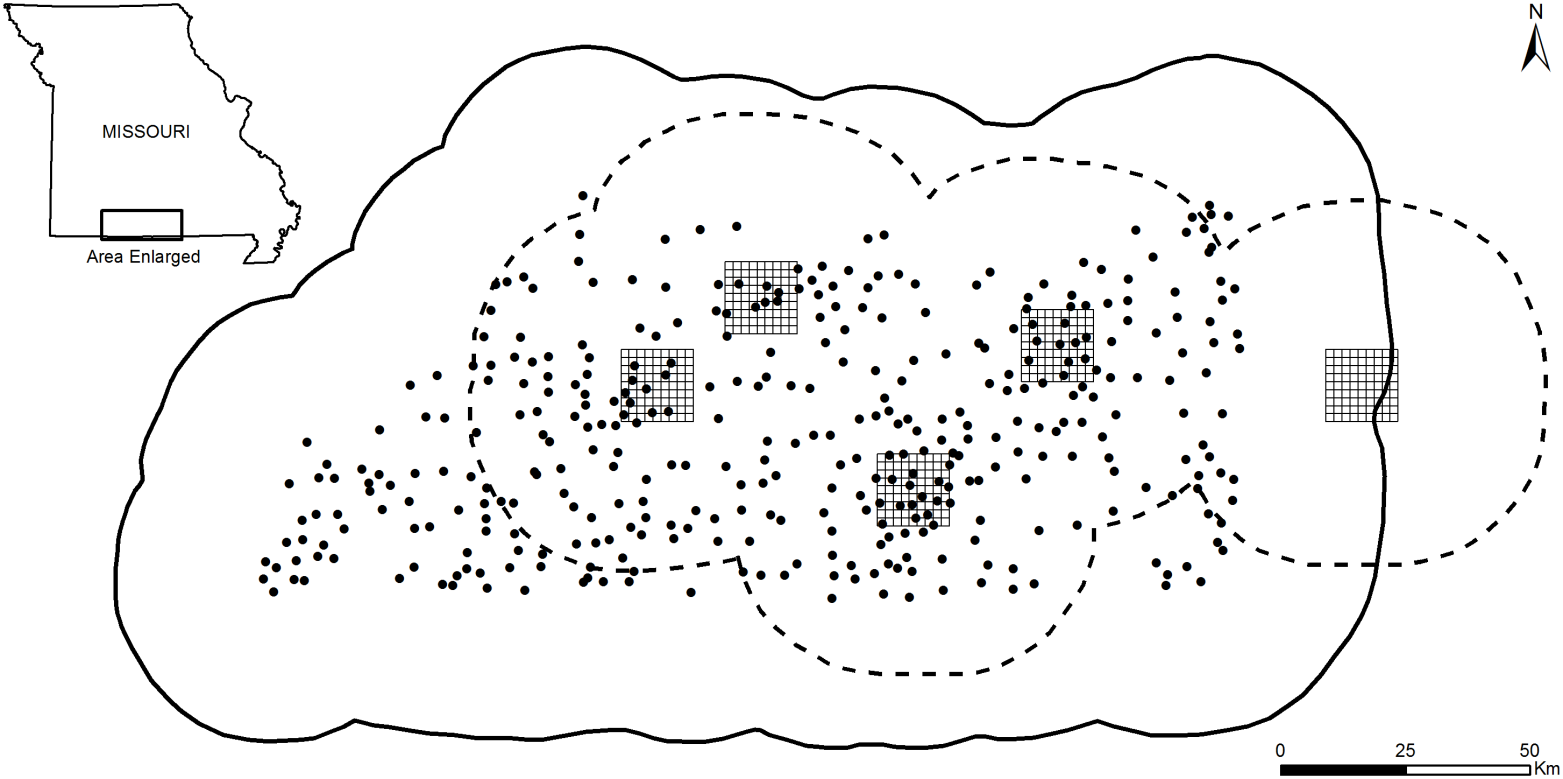
Trap array configurations. Location of the extensive and intensive configurations to estimate black bear density in south-central Missouri, USA. State space boundary for extensive (solid line) and intensive (dotted line) configurations represents the area used to estimate population size. For the extensive design, snares (black circles) were allocated proportionate to density of historical bear sightings. For the intensive design, five arrays were distributed in areas of expected bear occurrence and one snare was placed in each cell; specific locations omitted for clarity. The five arrays were designated alphabetically (A–E) from west to east.

We collected data from a recolonizing black bear population in the south-central Ozark Highlands ecological region of Missouri (36°30′–37°13′N, 91°16′–93°52′W), USA (Figure 1). The Ozark Highlands comprise 52% of the state’s total area and contains 57% forest, 32% crop and pasture, 2% grassland, and 7% developed areas [37]. About 80% of forest in Missouri occurs in the Ozark Highlands and is primarily upland oak-hickory (*Quercus* spp., *Carya* spp.) and oak-pine (*Pinus* spp.; [38]). Landownership in the Ozark Highlands includes private homesteads, farms, and public lands (e.g., Mark Twain National Forest, Ozark National Scenic Riverways). Elevation in Missouri ranges from 70 to 540 m with greatest elevations in the Ozark Highlands [39], [40].

### Data collection

We collected black bear hair samples using barbed-wire hair snares [41]. Snares were constructed using 4-barbed, 15.5 gauge wire to create an enclosure (∼1.5 m radius) around three or more trees. For the extensive design, we constructed snares using a single strand of barbed-wire set 50 cm above ground. For the intensive design, we constructed snares using two strands of barbed-wire with strands 20 and 50 cm above ground. We applied raspberry oil (intensive design only; Mother Murphy’s Laboratories, Inc., Greensboro, NC), anise oil (Minnesota Snareline Products, Pennock, MN), and Ultimate Bear Lure (Wildlife Research Center, Ramsey, MN) on perimeter trees forming the enclosure, about 2 m above ground. We placed decaying logs in the center of the enclosure and saturated them with 0.5 L of fish oil as an attractant [19]. Hair snare stations were re-lured every 10 days at the beginning of each consecutive sampling session. We collected hair samples at the end of each session and considered all hair found on a barb or single tree one sample. We placed hair in separate coin envelopes, and air dried samples before processing.

### Extensive sampling design

We derived the sampling boundary from a 70% fixed kernel isopleth applied to historical bear sightings (1989–2010) reported to the Missouri Department of Conservation by citizens throughout the state [42]. This area comprised 13,508 km^2^ in south-central Missouri. We used the 70% isopleth because this was the maximum logistically feasible extent of sampling and contained the core area of interest by state biologists. We used an array with 10×10 km cells to generate a distribution of bear sightings per array cell, excluding cells with zero bear sightings. We allocated 378 snares proportionate to the number of sightings per cell (Figure 1) following methods similar to Dreher et al. [18]. Cells containing 1–3 bear sightings received one snare, cells containing 4–5 sightings received four snares, cells with 6–7 sightings received five snares, and cells with >8 sightings received six snares. Cells with suitable habitat (i.e., forest) containing zero sightings adjacent to cells with similar habitat containing bear sightings were allocated snares similar to adjacent cells. We selected hair snare locations based on habitat characteristics and availability of forested private and public land. We used ArcMap 9.3.1 (Environmental Systems Research Institute, Redlands, California, USA) to select approximate locations for hair snares using forest cover data (30 m resolution, [43]) as initial criteria to maximize bear detection; excluding open water, agricultural, and developed areas.

We selected final snare locations within about 300 m of initial locations and out of sight from human trails or dwellings. Additionally, we used previous bear sightings, recent bear activity, and habitat and topographic features to select hair snare locations to maximize black bear capture [18]. We attempted to maintain a minimum distance of 3 km between snare sites, and conducted oversampling of snare locations in the event existing land use or ownership precluded snare placement. We monitored snares over six, 10-day sessions during June–August 2011.

### Intensive sampling design

We established 403 hair snares in five, 9×9 sampling arrays (A–E) with 2.6 km^2^ cells (210 km^2^/array) in south-central Missouri (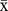 = 81 snares/array, SD = 1; Figure 1). We selected array locations to maximize coverage of the largest available forested regions known or expected to contain bears based on information from our extensive sampling effort and prior radio-collaring efforts [28]. Four arrays (A–D) were contained within the previous extensive survey area and array E was about 15 km east of the extensive survey boundary (Figure 1). We allocated one hair snare to each cell and monitored all snares over six, 10-day sessions during June–August 2012. Snares were set in forests on both private and public lands. We selected preliminary and final snare sites following the same criteria as the extensive sampling protocol.

### DNA extraction and microsatellite genotyping

We cut follicles from the shafts of hair to minimize the amount of melanin, a known PCR inhibitor [44], within each DNA extract. Number of follicles per sample ranged from one to twelve depending on amount of hair removed from each barb. We placed follicles in 1.5 mL Eppendorf tubes before adding 250 µL InstaGene matrix (BioRad, Hercules, CA). Samples incubated overnight at 56°C, then at 100°C for 15 min [45]. Following centrifugation at 13 krpm for 3 min, we transferred supernatant to a clean 1.5 mL tube before use in downstream reactions.

We genotyped samples at 15 microsatellite loci (G1A, G10B, G10C, G1D, G10J, G10L, G10M, G10O, G10P, G10U, UarMU05, UarMU10, UarMU23, UarMU59, and P2H03; [46]–[48]) following the protocol of Puckett et al. [49], where UarMU05 and UarMU10 were added to the multiplex panel in 2012 (Dataset S1). We randomly selected 25 samples genotyped at all loci to calculate the probability of identity between siblings (PID_sib_; α = 0.001) in Gimlet [50]. When determining recaptures we allowed two mismatches between samples. We determined the sex of unique individuals by amplification of the Amelogenin gene followed by BslI digestion [51].

### Population analysis

We used DNA-based encounter history data from hair snares and SCR models to estimate black bear density in south-central Missouri. We analyzed data using package *secr* (version 2.7.0; [52]) in program R [53]. We fit each model using a binomial observation model with the half-normal detection function, where the parameter g_0_ is the probability of detection at the activity center of an individual and σ is the spatial scale parameter of the detection function [54]. The spatial scale parameter describes the rate of decrease in capture probability as a function of increasing distance from a trap and an individual’s activity center [32]. We compared 12 *a priori* models for each array configuration (Table 1). We fit a null model with no covariates and 11 models with varying effects on the detection parameters (g_0_, σ). We created models based on expected sources of variation in black bear detection probability within our study area. Models with effects on g_0_ included one model with time as a factor (t), and three models with behavioral responses following initial detection (global learned response (b), snare-specific learned response (bk), and a snare-specific Markovian response (Bk)). We also fit seven models using sex as a categorical individual covariate to specify sex-specific effects on g_0_ and σ and in combination with the behavioral response models.

**Table 1:** Model selection results for fitted models ranked by AIC_c_ with number of parameters (*K*), log likelihood (LL), and AIC_c_ weights (*w_i_*) to estimate black bear density in south-central Missouri, USA, for extensive and intensive sampling designs.

Design	Model	*K*	LL	AIC_c_	ΔAIC_c_	*w_i_*
**Extensive**	g_0_(bk), σ(.)	4	−254.5	519.0	0.0	0.5
	g_0_(bk), σ(sex)	5	−253.6	520.3	1.4	0.3
	g_0_(Bk), σ(.)	4	−255.9	521.8	2.8	0.1
	g_0_(Bk), σ(sex)	5	−254.9	523.0	4.0	0.1
	g_0_(sex), σ(sex)	5	−266.6	546.4	27.4	0.0
	g_0_(.), σ(.)	3	−274.0	555.1	36.1	0.0
	g_0_(.), σ(sex)	4	−272.7	555.4	36.4	0.0
	g_0_(b), σ(.)	4	−273.2	556.4	37.4	0.0
	g_0_(b), σ(sex)	5	−271.8	556.7	37.8	0.0
	g_0_(sex), σ(.)	4	−273.7	557.3	38.3	0.0
	g_0_(t), σ(.)	8	−273.6	572.1	53.1	0.0
	g_0_(t), σ(sex)	9	−272.3	574.6	55.6	0.0
**Intensive**	g_0_(bk), σ(.)	4	−1175.8	2360.0	0.0	0.8
	g_0_(bk), σ(sex)	5	−1175.8	2362.2	2.2	0.2
	g_0_(Bk), σ(.)	4	−1215.7	2439.9	79.9	0.0
	g_0_(Bk), σ(sex)	5	−1215.2	2441.0	81.0	0.0
	g_0_(b), σ(.)	4	−1246.6	2501.8	141.8	0.0
	g_0_(sex), σ(.)	4	−1246.9	2502.3	142.3	0.0
	g_0_(.), σ(.)	3	−1249.0	2504.3	144.3	0.0
	g_0_(t), σ(.)	8	−1245.0	2507.7	147.7	0.0
	g_0_(sex), σ(sex)	5	−1299.5	2609.6	249.7	0.0
	g_0_(.), σ(sex)	4	−1301.0	2610.6	250.6	0.0
	g_0_(b), σ(sex)	5	−1300.9	2612.5	252.5	0.0
	g_0_(t), σ(sex)	9	−1296.7	2613.6	253.6	0.0

We fitted models using the half-normal detection function with baseline capture probability (g_0_) and scale parameter (σ). Effects on g_0_ and σ included time as a factor (t), global learned response (b), snare-specific learned response (bk), and a snare-specific Markovian response (Bk), and sex. Parameters with “.” indicate no effect.

We defined the state space (i.e., area of integration) as the area encompassing snares and all individuals potentially exposed to capture [30]. This area defines the extent of the distribution of home range centers in the population. We used three times the estimated σ to calculate the state space radius around snares [32] and tested if this was large enough using the mask check function in package *secr*. The state space radius was 45 km for the extensive design and 30 km for the intensive design, resulting in 41,121 km^2^ and 16,812 km^2^ areas, respectively. To estimate population size, we used the expected population size (*E(N)*) derived from the top supported model [34]. For the extensive design, we estimated population size using a 30 km radius (29,898 km^2^), as this represented our area of interest. We compared precision of parameter estimates using coefficients of variation (CV). We selected the top supported model for each configuration using Akaike’s Information Criterion corrected for small samples (AIC_c_) and considered models competing if within 2 AIC_c_ units from the top supported model [55].

### Simulations

We used our field sampling designs to simulate spatial capture-recapture datasets to evaluate accuracy of density estimates under each sampling configuration. We chose density and detection parameter values to represent both the observed values in our study and values commonly observed in other black bear studies in the United States [12], [56]–[58]. For density, we used values of 1.0 and 2.5 individuals per 100 km^2^, and capture probability (g_0_) values of 0.1 and 0.2. We also tested the scale parameter (σ) at 5, 10, and 15 km to investigate the effect of varying σ on density between sampling designs. We used a state space radius of 30 km when σ was 5 and 10 km and a radius of 45 km when σ was 15 km, with default point spacing of 64×64 points. Number of sampling intervals was set at six sessions. We then ran 100 replicates for each combination of density and detection parameter values (n = 12) under both configurations. For each scenario, we fit SCR models using the half-normal detection function in program DENSITY v5.0 [59]. To compare accuracy of density estimates to the true values from each sampling configuration we assessed average percent relative bias (%RB) and proportional coverage of confidence intervals (%COV).

## Results

### Microsatellite genotyping

To reduce genotyping error, we genotyped each hair sample three times before calling a consensus genotype [49] and we calculated an allelic dropout of 2.4% in Gimlet v1.3.3 [50] on 20 randomly selected samples. The probability of identity between siblings was 7.18×10^−4^; this level of PID_sib_ required samples to be genotyped at eight loci for inclusion in the study and required a genotyping rate of 61.5% for the extensive design and 53.3% for the intensive design to be included in the sample. Following removal of samples that genotyped at fewer than eight loci, in the extensive design there were 42 unique hair samples with an average genotyping rate of 93.8% across loci. In the intensive design, 224 unique hair samples were genotyped with an 83.8% genotyping rate.

### Extensive sampling design

We collected 98 black bear hair samples suitable for DNA extraction over six sessions from 30 unique snares (8% of the total number of snares). Number of black bear hair samples declined over time (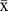 = 16.3, 28 samples in session one to eight samples in session six). Mean distance of each snare to nearest neighbor was 3.6 km (SD = 0.04 km). Mean distance between consecutive detection locations was 9.6 km.

Microsatellite marker analysis of hair samples revealed 25 unique individuals (11 F, 14 M) detected at 7% of all snares monitored (Dataset S2). Total detections per session ranged from six to nine (SD = 1.1), with 42 total detections, including within-session recaptures (Table 2). Individuals were detected on average 1.7 times (range = 1–5, SD = 1.0). Females were detected on average 1.9 times (range = 1–5, SD = 1.3) and males 1.5 times (range = 1–3, SD = 0.7). Fourteen individuals were not recaptured, including 55% of females and 57% of males. We detected individuals at an average of 1 snare (SD = 0.5, max = 2). Snares with ≥1 detection per session remained about constant (range = 6–8) across sampling sessions.

**Table 2:** Summary of sampling statistics for extensive and intensive (arrays A–E) black bear survey configurations in south-central Missouri, USA.

Design	Array	Snares^a^	u^b^	n^c^	Detections^d^	Snares Visited^e^	No. Hair Samples
**Extensive**		378	4.2 (2.4, 25)	6.5 (1.4, 39)	7.0 (1.1, 42)	6.8 (0.8, 26)	16.3 (7.2, 98)
**Intensive**	A	81	0.7 (0.8, 4)	0.8 (0.8, 5)	1.0 (0.9, 6)	1.0 (0.9, 4)	2.2 (2.3, 13)
	B	79	8.0 (6.7, 48)	14.7 (5.1, 88)	18.5 (7.0, 111)	12.8 (3.6, 36)	39.5 (18.4, 237)
	C	81	1.2 (0.8, 7)	1.8 (0.8, 11)	2.8 (1.2, 17)	2.7 (1.2, 10)	7.3 (5.7, 44)
	D	81	3.7 (2.9, 22)	8.0 (2.3, 48)	11.8 (2.7, 71)	9.2 (1.8, 29)	32.8 (12.0, 197)
	E	81	1.8 (1.8, 11)	2.7 (2.0, 16)	3.2 (2.2, 19)	2.5 (1.9, 12)	6.2 (4.4, 37)
**Intensive Total**		403	3.1 (2.6, 92)	5.6 (2.2, 168)	7.5 (2.8, 224)	5.6 (1.9, 91)	17.6 (8.6, 528)

Order of values are mean (standard deviation, total) over six sessions. Note the sum of new detections (u) was 92 total individuals for the intensive design due to two individuals being detected in two arrays (i.e., total individuals was actually 90).

a Number of lured snares in each session.

b Number of individuals detected for the first time on each session.

c Number of individuals detected on each session.

d Number of detections, including within-session recaptures.

e Number of snares having at least one detection per session.

**Figure 2:**
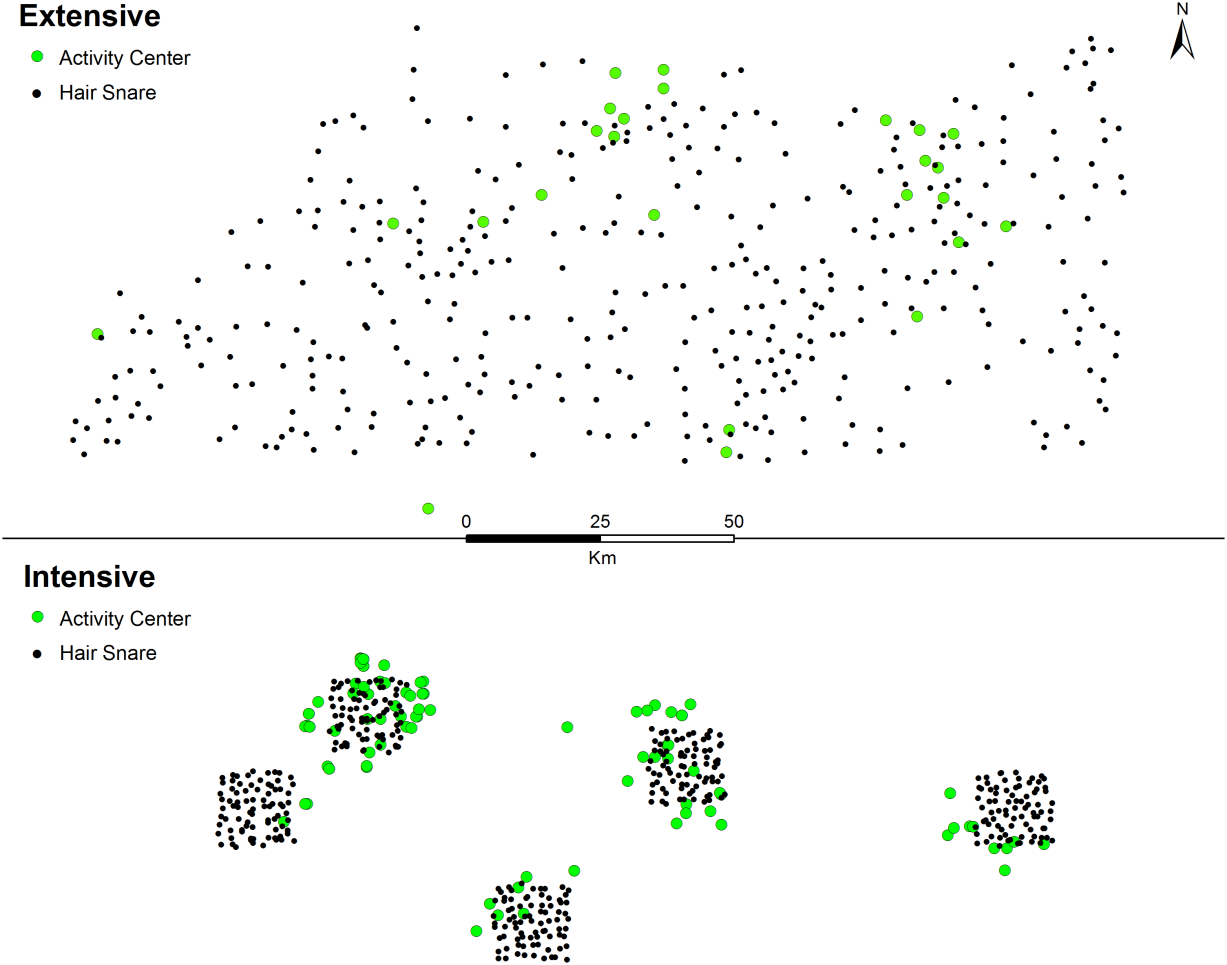
Estimated black bear activity centers. Location of hair snares and estimated activity centers (i.e., home range center) of identified bears with the extensive and intensive configurations in south-central Missouri, USA.

The snare-specific learned response model (bk) was most supported and 1.7 times more supported than the single competing model (g_0_[bk], σ[sex]; Table 1). However, we used parameter estimates from the top model as results between competing models were similar. Capture probability (g_0_) under the top model increased following initial detection at the same snare (Table 3). The estimated scale parameter of the detection function (σ) was 14.8 km (95% CI = 10.2–21.6 km). Density was 0.3 bears per 100 km^2^ (95% CI = 0.2–0.6). Expected population size (*E(N)*) was 91 individuals (SE = 31, 95% CI = 47–175) in our 29,898 km^2^ area of interest. Coefficients of variation (CV) ranged from 19% to 43% for all parameters. Activity centers estimated from the model were located primarily in the north-central and eastern portions of the trap array (Figure 2).

**Table 3:** Real parameter estimates and their precision (CV) for the most supported models to estimate black bear density (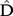; bears per 100 km^2^) for extensive and intensive array configurations in south-central Missouri, USA.

	Density				g_0_			σ		
Design	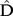	SE	95% CI	CV	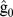	SE	CV	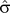	SE	CV
**Extensive**	0.3	0.1	0.2–0.6	34	bk_0_: 0.003	0.001	43	14.8	2.9	19
					bk_1_: 0.168	0.059	35			
**Intensive**	1.7	0.3	1.1–2.4	19	bk_0_: 0.011	0.002	15	8.5	0.9	10
					bk_1_: 0.166	0.025	15			

Capture probability (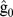) given for initial capture (bk_0_) and for previously captured individuals (bk_1_). Scale parameter of the detection function (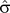) reported in kilometers.

### Intensive sampling design

We collected 528 black bear hair samples suitable for DNA extraction over six sessions from 110 unique snares (27% of total). Number of black bear hair samples collected in each array per session was variable (mean range = 2.2–39.5, grand mean = 17.6) and the total number of black bear hair samples collected declined over time (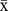 = 88.0; 107 samples in session one to 53 in session six). Mean distance of each snare to nearest neighbor within arrays was 1.0 km (SD = 0.04 km). Mean distance between consecutive detection locations was 2.5 km.

Microsatellite marker analysis of hair samples from snares revealed 90 unique individuals (59 F, 31 M; 4–48 individuals/array) detected at 23% of all snares monitored (Table 2; Dataset S2). Total detections per session ranged from 25 to 43 (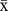 = 37.3, SD = 7.5), with 224 total detections, including within-session recaptures. Individuals were detected on average 2.5 times (range = 1–10, SD = 2.5). Females were detected on average 2.6 times (range = 1–10, SD = 2.7) and males 2.3 times (range = 1–10, SD = 2.2). Fifty-five individuals were not recaptured, including 63% of females and 61% of males. Two individuals were detected in two arrays, a male with an inter-trap distance of 57 km and a female with an inter-trap distance of 31 km. We detected individuals at an average of 2.0 snares (SD = 1.6, max = 8), and number of snares having ≥1 detection varied among arrays (range = 4–36; Table 2).

The snare-specific learned response model (bk) was most supported and four times more supported than the next best model (g_0_[bk], σ[sex]; Table 1). Capture probability under the top model increased following initial detection at the same trap, and σ was 8.5 km (95% CI = 7.0–10.4 km; Table 3). Density of the pooled arrays was 1.7 bears per 100 km^2^ (95% CI = 1.1–2.4). Expected population size (*E(N)*) was 279 individuals (SE = 54, 95% CI = 193–406) in the 16,812 km^2^ state space. Coefficients of variation ranged from 10% to 19% for all parameters. Estimated activity centers were primarily clustered within and around arrays B and D (Figure 2).

Since we only added a lower strand of barbed wire to hair snares in the intensive configuration, we also describe detections after excluding hair samples captured on the lower strand to compare sample size and distribution of detections to the full data set. Of the 224 total detections, 35 (16%) resulted from hair samples collected from the bottom strand and represented 30 individuals (23 females, 7 males). Exclusion of these detections resulted in the loss of 14 unique bears (10 females, 4 males) from the data set. Bottom strand detections also resulted in the loss of 11 observed movements (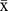 = 7.7 km, SD = 16.3) between successive recaptures at different snares, including the largest distance (57 km) moved between arrays (B and D) by a male bear, which was also the largest movement in the full data set.

### Simulations

For our low density simulations (1.0 bear per 100 km^2^) with σ<15 km, both array configurations had relative bias less than ±2.0% and 95% confidence interval coverage ≥92% (Table 4). Similarly, at 2.5 bears per 100 km^2^ both configurations produced almost unbiased density estimates at both g_0_ levels and when σ<15 km. At σ = 15 km, relative bias for the extensive design remained similar to other scenarios, but showed a positive increase for the intensive design. Both configurations performed well when simulation scenarios reflected field results of each configuration.

**Table 4:** Percent relative bias (%RB) and percent coverage of 95% confidence intervals (%COV) of mean density estimates 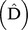 for simulations of spatial capture recapture models under extensive and intensive trap configurations.

			Array Configuration							
Scenario			Extensive				Intensive			
D	g_0_	σ	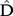	SE	%RB	%COV	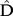	SE	%RB	%COV
1.0	0.1	5	1.01	0.09	1.21	92	1.00	0.16	−0.01	93
		10	0.99	0.07	−0.72	96	0.99	0.09	−0.84	94
		15	1.00	0.13	0.07	93	1.01	0.07	0.66	97
	0.2	5	1.00	0.08	−0.08	97	1.00	0.14	−0.28	94
		10	1.00	0.07	−0.18	97	1.02	0.09	1.94	96
		15	1.00	0.13	−0.26	96	1.03	0.07	3.43	93
2.5	0.1	5	2.47	0.01	−1.01	95	2.50	0.03	−0.01	90
		10	2.49	0.01	−0.45	95	2.51	0.02	0.28	91
		15	2.49	0.09	−0.22	98	2.53	0.11	1.36	96
	0.2	5	2.51	0.13	0.58	92	2.49	0.02	−0.52	97
		10	2.50	0.01	−0.01	92	2.53	0.01	1.22	92
		15	2.51	0.12	0.59	96	2.57	0.11	2.62	91

Estimates are based on averages over 100 replicates for each scenario of density (1.0, 2.5 bears per100 km^2^), g_0_ (0.1, 0.2), and σ (5, 10, 15 km).

## Discussion

We found that multiple arrays spaced across a landscape using intensive snare coverage yielded more captures and recaptures of more individuals than extensive coverage spaced over an area approximately 13 times larger. Consequently, estimated density using the intensive configuration was 5.5 times greater than that under the extensive configuration. By pooling detections among our arrays with closer snare spacing and using SCR models to explicitly account for variable exposure to traps, we were able to increase precision while retaining the ability to estimate average density over a landscape [60]–[62]. However, placement of intensive arrays was informed largely by the distribution of detections from the extensive sampling effort. Therefore, although results support the intensive design, prior knowledge of bear distribution was critical to increasing detections in the intensive configuration. When population distribution and space use are poorly understood, adjusting sampling design over multiple surveys may be required [28].

With intensive sampling, CV of parameter estimates decreased on average by 53% compared to extensive sampling. Pollock et al. [63] recommended a CV<20% for reasonable precision of estimates, which we achieved for all parameters with our intensive configuration. Boulanger et al. [28] also demonstrated increased capture probability and precision under intensive sampling of a grizzly bear (*Ursus arctos*) population. However, over 50% of individuals in our study were not recaptured under both sampling designs and capture probability remained below recommended levels (i.e., >0.2; [28]). The lack of food reward at snares, summer migration to find food, mating opportunities, or dispersal [64], [65], may partially explain low recaptures observed during our summer (June–August) surveys. We suggest greater precision under the intensive design was largely due to detecting a greater proportion of individuals and increased detections at multiple snares [28], [29]. Though we cannot discern potential demographic changes between years, it is important to consider potential year effects on parameter estimates. For example, seasonal food abundance can affect movements and responses to baited sites [64], [66]. However, model selection results suggest bears responded to lured snares similarly between years. Therefore, we suggest our comparisons are appropriate given the constraints of implementing such large scale capture-recapture studies.

Although our two sampling designs are not comparable experimentally, results demonstrate the interplay among spatial sampling design, population distribution, and precision of detection parameters [35], [60]. The extensive configuration covered a wide geographic area, but snare distribution either covered large areas of unoccupied habitat or snare spacing was too wide given individual movements [29]. Low precision of parameter estimates with extensive sampling may illustrate the reality of simulations by Tobler and Powell [27], where precision decreased as trap spacing increased with larger array size. The logistical constraints of implementing such a large survey and risk of obtaining insufficient detections makes this approach unattractive for low density populations, especially those unevenly distributed over the landscape. This sampling design has proved effective in populations with higher bear density and larger home range size when population estimation was combined with independent data from hunter harvests [18]. In less studied and non-harvested populations, such as in Missouri, auxiliary information is often unavailable or too cursory to accurately inform study design or analyses.

Detections over the extensive and intensive arrays were not uniformly distributed, with most detections concentrated in two distinct areas during both years (Figure 2). If the low detection areas of the extensive design resulted from insufficient sampling alone, we would expect detections to increase in these areas when using intensive sampling [67], assuming minimal demographic changes between years. Although overall detections were greater using the intensive design, two of the four arrays that overlapped the extensive design area still received low detections. Though ancillary, this spatial pattern of detections during both years suggests a low, heterogeneous density as opposed to insufficient sampling design. Moreover, Karanth et al. [68] demonstrated a positive relationship between spatial coverage of traps and total animals detected. With extensive sampling, we detected 25 bears over a nominal array area of about 13,500 km^2^ and with intensive sampling we detected 90 bears over about 1,000 km^2^. That our results were not consistent with findings by Karanth et al. [68] further suggests a population where most individuals occurred in clustered regions with few bears interspersed between these areas. Heterogeneous densities are common among large carnivore populations in a varied landscape [58], [62], particularly among recently recolonizing populations [12], [49], [69].

Changes to the intensive design, including the addition of a lower strand of barbed wire, increased the number of unique individuals and overall detections. Excluding lower strand detections greatly affected the number and spatial distribution of detections. One of the male detection losses represented the largest detected movement (57 km) between snares. Sex-specific space use can bias detection in carnivore population surveys [15], and our results illustrate the potential importance of spatial sampling design and snare design to increasing overall detections and sex-specific movements among snares. Some studies have attempted to quantify the effectiveness of using a second, lower strand of barbed wire to increase capture probability or identify family groups [18], [28]. Whereas Boulanger et al. [70] found that a lower strand did not greatly affect estimates for a grizzly bear population, we contend that for low density populations a second strand may sufficiently increase data on encountered individuals and movements.

Simulations of our extensive and intensive configurations showed low bias and adequate confidence interval coverage for all scenarios. Although bias was low, the positive bias for the intensive design when σ = 15 km suggests increasing distance between snares to extend spatial coverage may increase the likelihood of detecting large movements [35]. Increasing spacing in the intensive design likely wouldn’t affect precision of σ as our effective trap spacing (i.e., spacing/σ; [29]) was much narrower than the <2σ suggested by Sun et al. [29]. Whereas both designs had low bias in simulations, few detections and inter-trap recaptures precluded precise density estimation for the extensive design field study, though precision of σ remained adequate (i.e., CV<20%). These results show that although SCR models are robust to variable spatial sampling designs [29], [35], in low density populations, or populations with non-uniform space use, optimizing the tradeoff between snare spacing, coverage, and sample size is critical for estimating σ and density with high precision and accuracy.

Management decisions for large mammals are typically made over large spatial scales [18], [71], [72], and inferences informing these decisions should cover a similar area [73]. However, logistical constraints and carnivore ecology often preclude large scale inference [74], [75]. For example, although SCR may be robust to our extensive sampling design, low detections still hindered precise density estimation over such a large region [35], [63]. Moreover, increasing trap intensity over a smaller region poses limits to the extent of density extrapolation to a larger area [74]. Thus, our extrapolation of density to a population estimate of 279 (95% CI = 193–406) black bears in a 16,812 km^2^ area must be treated with caution [74]. This estimate also cannot be compared to that from extensive sampling as they were derived from different areas. However, given knowledge of bear presence and movements within this region (J. Beringer, unpublished data), the location of our intensive arrays likely sampled a representative range of bear densities and focused on areas with known populations [60].

## Conclusion

Our study highlights important considerations in sampling design for attaining precise estimates using SCR models for wide-ranging mammals. Although SCR models are flexible to various spatial designs [34], they remain sensitive to the number of detections and inter-trap recaptures across the range of individual movements [35]. Complete spatial coverage with sufficient trap spacing is challenging when animals with large home ranges exist at low densities and are not uniformly distributed [35], [62], [76]. Given these conditions, multiple arrays with intensive trap spacing similar in extent to individual movements should increase precision of detection parameters. We demonstrated support for sampling recommendations from simulated SCR analyses of black bear data sets [29], [35] and illustrated realistic challenges of tailoring large scale spatial trap designs to a species’ behavior and spatial ecology. Although our study was specific to black bears, we suggest our findings are applicable to other wide ranging and low density species. The flexibility of SCR models to various sampling designs and techniques provide increased opportunities to accurately survey rare and elusive animals of high management or conservation priority.

## Supporting Information

Dataset S1 **Microsatellite genotypes of individual bears sampled at hair snares.**
(XLSX)

Dataset S2 **DNA encounter histories from extensive and intensive design field seasons.**
(XLSX)
